# Prenatal stress and fluoxetine exposure in BTBR and B6 mice differentially affects autism-like behaviors in adult male and female offspring

**DOI:** 10.1016/j.physbeh.2025.114891

**Published:** 2025-03-29

**Authors:** Anna L. Arzuaga, Pamela Teneqexhi, Katelyn Amodeo, John R. Larson, Michael E. Ragozzino

**Affiliations:** a Department of Biological Sciences, University of Illinois Chicago, Chicago, IL 60607, USA; b Department of Psychology, University of Illinois Chicago, Chicago, IL 60607, USA; c Department of Psychiatry, University of Illinois Chicago, Chicago, IL 60612, USA

**Keywords:** BTBR, Prenatal stress, SSRI, Learning, Behavioral flexibility, Repetitive behaviors

## Abstract

Autism spectrum disorder (ASD) is characterized by significant heterogeneity in the variety and severity of symptoms. Prenatal stress and/or exposure to antidepressants may be major contributors to ASD heterogeneity. To date, the effects of prenatal stress or selective serotonin reuptake inhibitor exposure have been primarily examined in common laboratory rat and mouse strains as opposed to in rodent models of autism. The present experiments determined in the BTBR mouse model of autism whether restraint stress (30 min session every 2 days during G4 - G18) and/or exposure to the SSRI, fluoxetine (3 mg/kg during G8 - G18) affects repetitive motor behaviors, anxiety and/or behavioral flexibility in offspring at adulthood. Male and female BTBR mice exhibited elevated grooming behavior compared to that of C57BL/6 J (B6) mice. The prenatal manipulations did not affect grooming in male BTBR mice, but the combination increased rearing and jumping. Prenatal stress, fluoxetine and the combination significantly reduced self-grooming, while concomitantly increasing locomotion in female BTBR mice. These prenatal manipulations also increased rearing and jumping behavior in female BTBR mice. In B6 mice, the prenatal stress conditions increased grooming behavior. In addition, male BTBR mice exposed to prenatal stress and fluoxetine along with female BTBR mice prenatally exposed to fluoxetine were impaired on reversal learning. The prenatal manipulations had no effect on anxiety in either mouse strain. The pattern of results suggest that prenatal exposure to stress and/or a SSRI have long-term effects on autism-like behaviors and may contribute to the heterogeneity and co-morbidity observed in autism.

## 1. Introduction

Over the past two decades, the prevalence of autism spectrum disorder (ASD) has increased from 1 in 150 to 1 in 36 [1]. Various studies indicate that maternal stress during pregnancy is associated with increased risk for autism spectrum disorder (ASD) in offspring [2–5]. Similar to the increase in autism prevalence, antidepressant use in pregnancy has significantly increased, with selective serotonin reuptake inhibitors (SSRI) the most commonly prescribed [6,7]. Because serotonin plays an important role in brain development, perturbations in the serotonin system during the prenatal period could have long-lasting effects on brain and behavioral function [8–10]. Numerous studies have examined whether maternal stress, depression and/or SSRI use during pregnancy increases risk for ASD in offspring resulting in varied outcomes. Some studies suggest maternal stress, depression and/or SSRI use during pregnancy increases risk [11,5]. Other findings suggest that SSRI use during pregnancy does not increase risk at least when compared with pregnant women with a psychiatric condition not treated with a SSRI [12–17]. Alternatively, the possibility that depression and/or SSRI use prior to conception may be more predictive has been proposed [18]. While still other results suggest that prenatal SSRI exposure may be a greater risk factor for ASD in males, but not females [19]. As commonly noted in these studies there is a significant challenge in controlling for the numerous factors to clearly identify the contribution that maternal stress and/or SSRI use during pregnancy contribute to ASD risk. Thus, there is a difficulty in drawing a causal effect of maternal stress, SSRI treatment or the combination during pregnancy on offspring.

An alternative and complementary approach to the epidemiological studies is to systematically manipulate maternal stress and SSRI exposure during pregnancy in preclinical experiments. One recent study investigated the effects of prenatal SSRI exposure with escitalopram in Long-Evans rats on autism-like behaviors in offspring at adulthood [20]. Escitalopram administered to pregnant dams during the first half of gestation (G1-G10) reduced anxiety-like behavior and improved behavioral flexibility with no effect on social or repetitive motor behaviors in male and female offspring. Escitalopram exposure during the second half of gestation (G11-G20) increased repetitive motor behavior (marble burying), but did not affect other measures. In another study using B6 mice, the effects of prenatal stress and/or SSRI exposure with fluoxetine was examined in male and female offspring at adulthood [21]. Prenatal exposure to fluoxetine (G8-G18) alone or prenatal stress exposure alone (G4-G18) did not affect anxiety-like behavior or spatial learning with probabilistic reinforcement in male and female offspring. Further, prenatal fluoxetine or stress exposure did not affect probabilistic reversal learning in male offspring, but did impair probabilistic reversal learning in female offspring. In contrast, prenatal exposure to the combination of stress and fluoxetine significantly elevated repetitive motor behavior and impaired probabilistic reversal learning selectively in male offspring. Taken together, the studies suggest that prenatal exposure to SSRI alone has minimal behavioral effects on autism-like behaviors in offspring. Further, prenatal stress in mice also has minimal effects on autism-like behaviors in offspring, but the combination of prenatal stress and SSRI exposure increases autism-like behaviors in offspring, but preferentially in male offspring. However, these experiments were carried out in common strains of laboratory rats and mice as opposed to rodents that serve as models of autism. Investigating these factors in rodent models of autism can provide valuable insights into how prenatal stress and/or SSRI antidepressant exposure may have differential effects.

Heterogeneity in ASD is well-documented and may arise, in part, from an interaction of environmental and genetic risk factors [22,23]. Polygenic models suggest that ASD risk results from multiple inherited gene variants, that individually have small effects, but combined with environmental factors lead to significant risk [24–27]. Prenatal exposure to stress and/or SSRI antidepressants may be two factors that affect ASD risk and heterogeneity. The BTBR mouse is frequently referred to as an idiopathic model of ASD, but one study revealed that compared to that of B6 mice, BTBR mice have multiple gene mutations that have been observed in ASD individuals [28]. Thus, the BTBR mouse might also be considered a polygenic model of ASD. Furthermore, the BTBR mouse exhibits a range of social, communicative, repetitive motor and behavioral flexibility alterations comparable to that observed in ASD [29–35]. Taken together, the BTBR mouse may be a useful mouse model to investigate how prenatal stress and/or SSRI exposure affects behavioral outcomes in offspring.

The present experiment investigated whether prenatal stress and/or fluoxetine exposure during gestation affects autism-like behaviors in male and female offspring of BTBR and B6 mice (control strain).

## 2. Methods

### 2.1. Subjects

Male and female BTBR *T*+ Itpr3tf/J (BTBR) and C57BL/6 J (B6) mice bred in the laboratory served as subjects. Initial breeders were acquired from the Jackson Laboratory (Bar Harbor, ME). Mice were individually housed in plastic cages (28 cm wide × 17 cm long × 12 cm high) in a humidity (30–40 %) and temperature (23 °C) controlled room and were maintained on a 12 h light/dark cycle (lights on at 7:30 a.m.). Animal care and experiments were followed in accordance with the National Institute of Health Guide for the Care and Use of Laboratory Animals and were approved by the Institutional Laboratory Animal Care and Use Committee of the University of Illinois Chicago.

### 2.2. Prenatal stress exposure and prenatal fluoxetine treatment

Eight to twelve-week-old BTBR female mice were bred with age-matched BTBR male mice, as well as eight to twelve-week old B6 female mice bred with age-matched B6 male mice. Pregnancy was determined by the detection of a vaginal plug in the morning defined as gestation day 0 (GD0) and individually housed. Pregnant dams were randomly assigned to one of the following four conditions: 1) no restraint/vehicle; 2) no restraint/fluoxetine; 3) restraint/vehicle or 4) restraint/fluoxetine. In a past study [21], pregnant B6 mice received restraint stress for 3 – 30-minute sessions daily starting on G4 through G18. This same restraint stress protocol in BTBR mice dramatically reduced litter survival. In a subsequent pilot study, we compared the effects of restraint stress given for a single 30-minute session from G4 to G18 versus restraint stress given for a single 30-minute session every other day from G4 to G18. Because the latter protocol produced more viable litters (see Supplementary Figure 1), the experiment employed restraint stress for a single 30-minute session given every other day. The restraint stress protocol was administered to pregnant dams by physical restraint in a plastic tube (12 cm × 3 cm) for a single 30-minute session starting on gestation day (GD) 4 through GD 18. Because the restraint was administered every other day a pregnant mouse received a total of 8 restraint sessions. As in a previous study [21], dams in the no restraint condition were left undisturbed in their home cage. Mice from both the no restraint and restraint stress groups were randomly assigned to vehicle (sterile saline) or fluoxetine (3 mg/kg) treatment. From gestational day 8 until birth, pregnant dams were weighed and received a daily subcutaneous injection of either vehicle or 3 mg/kg fluoxetine (Bio-Techne Corporation, Minneapolis, MN). The fluoxetine dose was chosen based on previous studies in mice [21,36]. In B6 mice, the sample size for dams in each condition was as follows: No Restraint/Vehicle, *n*= 4; No Restraint/Fluoxetine, *n*= 6; Restraint/Vehicle, *n*= 4; Restraint/Fluoxetine, *n*= 6. In BTBR mice, the sample size for dams in each condition was as follows: No Restraint/Vehicle, *n*= 5; No Restraint/Fluoxetine, *n*= 3; Restraint/Vehicle, *n*= 4; Restraint/Fluoxetine, *n*= 5. Litter size at birth was measured. Litter sizes with a range of 2 to 12 pups were included in this behavioral study. The day of birth was designated as postnatal day 1 (P1). Pups were weaned at postnatal day (P21) and group housed with 2–4 littermates of the same sex until experimentation. Sample sizes for offspring ranged from *n*= 7–13.

### 2.3. Sucrose preference test

In a past study that involved a greater amount of restraint stress, a reduced sucrose preference in pregnant B6 mice was observed that was reversed by fluoxetine treatment [21]. We wanted to determine whether the reduced amount of restraint stress and fluoxetine in the present study still affected a sucrose preference in B6 mice. Although BTBR exhibit an altered discrimination of sweet vs. non-sweet substances due to a mutation in the inositol triphosphate receptor 3 gene [37], we conducted a sucrose preference test in BTBR mice to compare with B6 mice. A two-bottle paradigm was used in which pregnant dams had free access to a solution of 1 % sucrose solution for 12 h during their night cycle from G14-G16. On G14, females had free access to two bottles of 1 % sucrose solution to eliminate neophobia as a confounding variable. On G15 and G16, pregnant dams had free access to 1 % sucrose and water with bottle position switched across days. Sucrose consumption during the final test day session was calculated as the percentage of sucrose preference = 100 × [(sucrose intake during test day 3)/ (sucrose intake during test day 3 + water intake during test day 3).

### 2.4. Repetitive grooming behavior

All male and female offspring of B6 and BTBR dams from the four conditions were tested for spontaneous self-grooming at 7 weeks of age. Mice were individually placed in a clear plastic cage (28 cm wide × 17 cm long × 12 cm high) for a total of 20 min. The plastic cage was placed in a room separate from the mouse housing room. Subjects were free to explore the cage for the entirety of the test. To ensure the mice adjust to a novel environment, the first 10 min served as a habituation period. During the second 10 min of testing, cumulative time spent grooming was manually recorded by pressing a designated key on a computer connected to a USB camera with behavioral tracking software, ANY-maze (Version 6.3; Stoelting Co., Wood Dale, IL). Grooming behavior such as head washing, body grooming, paw/leg licking, and genital/tail grooming were included. The plastic cage was thoroughly cleaned with 2 % quatricide solution between tests.

### 2.5. Locomotor activity

Locomotor behavior was measured for all mice during the self-grooming test. In the same apparatus, subjects were allowed to freely explore the cage for the entirety of the 20 min test. The initial 10 min served as a habituation period in which mice acclimated to a novel environment. During the second 10 min of testing, measurement of total distance travelled in meters was automatically recorded by behavioral tracking software, ANY-maze (Version 6.3; Stoelting Co., Wood Dale, IL).

### 2.6. Rearing and jumping behavior

Observations during the self-grooming test indicated there were possible differences in rearing and jumping behaviors among strains and conditions. We did a subsequent analysis of rearing and jumping behavior based on the recorded self-grooming test videos. Rearing and jumping behavior was only measured during the second 10 min of the test similar to self-grooming. Rearing was defined as a mouse raising its two front limbs off the floor. Jumping behavior was defined as all four limbs raising off the floor. The number of jumps in a test session was computed, as well as the proportion of mice within a group that initiated a jump. One male BTBR mouse and one male B6 mouse each from the no restraint/vehicle condition were not included in the analyses because their videos became corrupted and could not be viewed.

### 2.7. Elevated plus-maze

Two days after the self-grooming test, mice were tested in an elevated plus-maze (Stoelting Company, Wood Dale, IL, USA). Mice received one 10-minute test. The elevated-plus maze (EPM) was modified from an eight-arm radial maze made of grey acrylic such that four arms. served as choice arms that formed the shape of a plus sign. The other four arms were blocked off. Each arm was 35 cm long and 5 cm wide. The two closed arms had walls (18 cm in height) and two open arms had no walls. The maze had a center area approximately 12 cm in diameter. The maze was elevated 70 cm above the floor.

### 2.8. Spatial discrimination test

#### 2.8.1. Spatial discrimination apparatus

Spatial discrimination training was conducted in a black acrylic rectangular maze (76 cm long × 50 cm wide × 30 cm high). A central wall extending the full width of the maze separated the start and choice areas. Access to the choice area was controlled by a small plastic door (10 cm high × 5 cm wide) located at the midpoint of the central wall. The choice area was divided into two equally sized spatial quadrants by an acrylic piece (30 cm long × 16 cm high) extended out from the back wall of the maze. Each spatial area contained a centered food well placed securely near the back of the wall and several distinct visual cues mounted on their corresponding back and side walls.

#### 2.8.2. Spatial discrimination training

The day after the elevated plus-maze test, mice were food restricted to 85 % of their ad libitum body weight. Once their weight was stabilized (~ a week later), training sessions were conducted 2–4 days before acquisition testing as described in previous studies [29,30,38]. Mice were placed in the start area of the maze for one minute prior to opening the small plastic door to acclimate. Once opened, mice were allowed to freely roam the choice area and consume a half piece of Fruity Pebbles cereal (Post Foods, St. Louis, MO) from each food well. After consumption, mice were trained to return to the start area and food wells were rebaited. Subsequent trials were performed until 15 min elapsed. Training criteria required mice to consume cereal pieces from each food well 6 or more times for two consecutive days before advancing to the acquisition phase.

#### 2.8.3. Acquisition and reversal learning

During acquisition trials, the food well in the correct choice area contained a half piece of Fruity Pebbles cereal while the food well in the other choice area never had a cereal piece. If a mouse chose the spatial location with a cereal piece, it was allowed to consume it and then return to the start area. If a mouse chose a spatial location without a baited food well it was allowed to explore the food well and then return to the start area. Only entries into the correct spatial location were scored as a correct choice. The choice areas were cleaned with 2 % quatricide solution between trials. The inter-trial interval was approximately 15 s.

A mouse reached criterion when it made six, correct consecutive choices as in past experiments. A retention test was administered the following day in which a mouse was required to choose the same location as on acquisition with a criterion of five out of six correct trials [29, 30]. After retention, mice were placed into their home cage and returned to their housing room. Thirty minutes following the retention test, mice were placed in the maze for the reversal learning test. All aspects of testing were the same as acquisition, except that the spatial location reinforced on acquisition was never reinforced and the other spatial location was always reinforced. Reversal learning criterion was achieved when a mouse completed six consecutive correct trials.

A few mice receiving the self-grooming and elevated-plus maze tests were subsequently tested on a spatial discrimination involving probabilistic reinforcement and not in the version described above. The final n’s for the mice groups tested in the spatial discrimination described above ranged from 7–12.

### 2.9. Statistics

A two-way ANOVA determined whether restraint stress, fluoxetine or combination affected B6 and/or BTBR pregnant dams’ weight gain, litter size and sucrose preference. Behavioral measurements in offspring were analyzed using ANOVA mixed linear models (R version 4.4.1) to control for litter size [39,40]. Strain, sex, condition and their interactions were fixed factors with litter size included as a random effect. Kenward-Roger adjusted degrees of freedom were used. The statistical significance level was defined at *p* <0.05. Tukey post-hoc tests were used to determine significant between group differences. Tests for difference in proportions were conducted for litter survivability among the female dam groups and for jumping behavior among the groups in offspring.

## 3. Results

### 3.1. Gestational weight gain, litter survivability and litter size

**Fig. 1.**
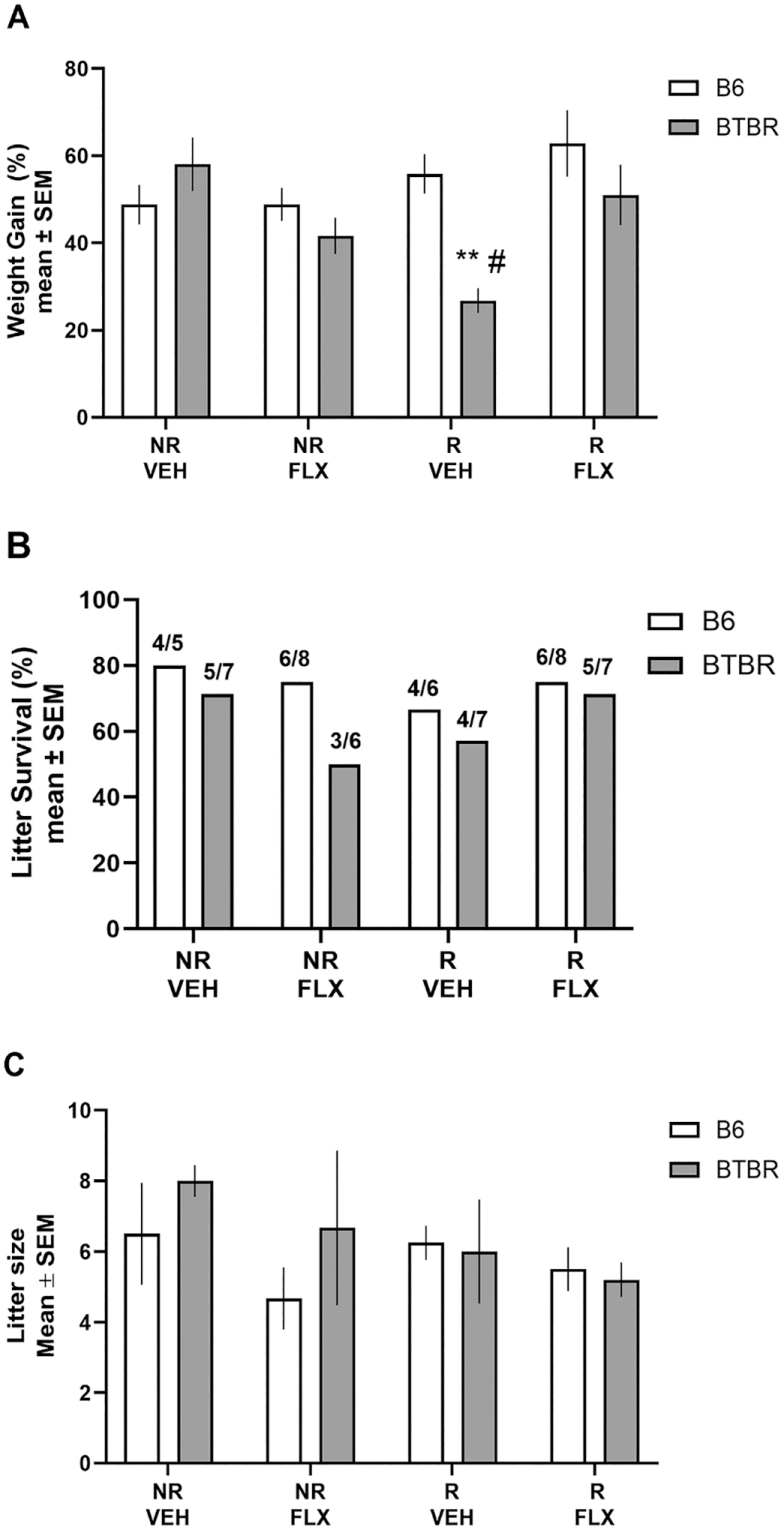
B6 and BTBR mouse weight gain during gestation, litter survivability and litter size. (A) Mean (± SEM) percent gestational weight increase. The ratio was calculated as the percentage of final weight recorded at the end of pregnancy (GD18) subtracted by starting weight on GD0/the absolute value of GD0 wt x 100. Restraint stress significantly reduced weight gain in BTBR mice; (B) Percent litter survival across strain and condition. There were no significant differences in litter survival (C) Mean (± SEM) litter across strain and condition. There were no significant differences in litter size. NR/VEH = no restraint/vehicle; NR/FLX = no restraint fluoxetine; R/VEH = restraint/vehicle and R/FLX = restraint/fluoxetine. # = *p*< 0.05 vs. B6 mice restraint stress/vehicle, BTBR mice restraint stress/fluoxetine; ** *p*< 0.01 vs. BTBR mice no restraint/vehicle.

Pregnant BTBR and B6 dams were weighed daily to determine whether restraint stress and/or fluoxetine treatment affected gestational weight gain. BTBR females in all groups started between 29–33 g while B6 females in all groups weighed between 20–23 g With the exception of the BTBR mice exposed to restraint stress, pregnant dams in both strains across the various conditions exhibited a 40–50 % increase in weight across gestation (see Fig. 1A). BTBR mice exposed to restraint stress only exhibited a 25–30 % weight gain across gestation that was reflected in a significant interaction (F_3,29_= 3.06, *p*= 0.044). Post-hoc tests revealed that the weight gain for BTBR mice exposed to restraint stress during pregnancy was significantly less than that of BTBR mice in the no restraint/vehicle condition (*p*< 0.01), as well as B6 mice exposed to restraint stress and BTBR mice exposed to restraint stress and fluoxetine during pregnancy (*p*’s < 0.05).

Litter survival rate was also measured (see Fig. 1B). In BTBR mice, fluoxetine treatment alone or restraint stress alone reduced litter survivability to ~50–60 % compared to the other conditions that had ~70 % survivability. In B6 mice, restraint stress alone or restraint stress/fluoxetine conditions led to ~60 % litter survivability compared to 80 % survivability in the other conditions. However, there were no significant differences between strains in the various conditions. In addition to litter survival rate, litter size was analyzed. Fig. 1C illustrates the mean litter size in each condition for BTBR and B6 mice. Although variable, the different conditions in both strains led to a comparable litter size. Analysis of litter indicated that there was not a significant main effect for strain (*F*_1,29_= 1.10, *p*= 0.30) or condition (*F*_3,29_= 1.48, *p*= 0.24), nor a significant interaction (*F*_3,29_= 0.72, *p*= 0.55).

### 3.2. Sucrose preference

**Fig. 2.**
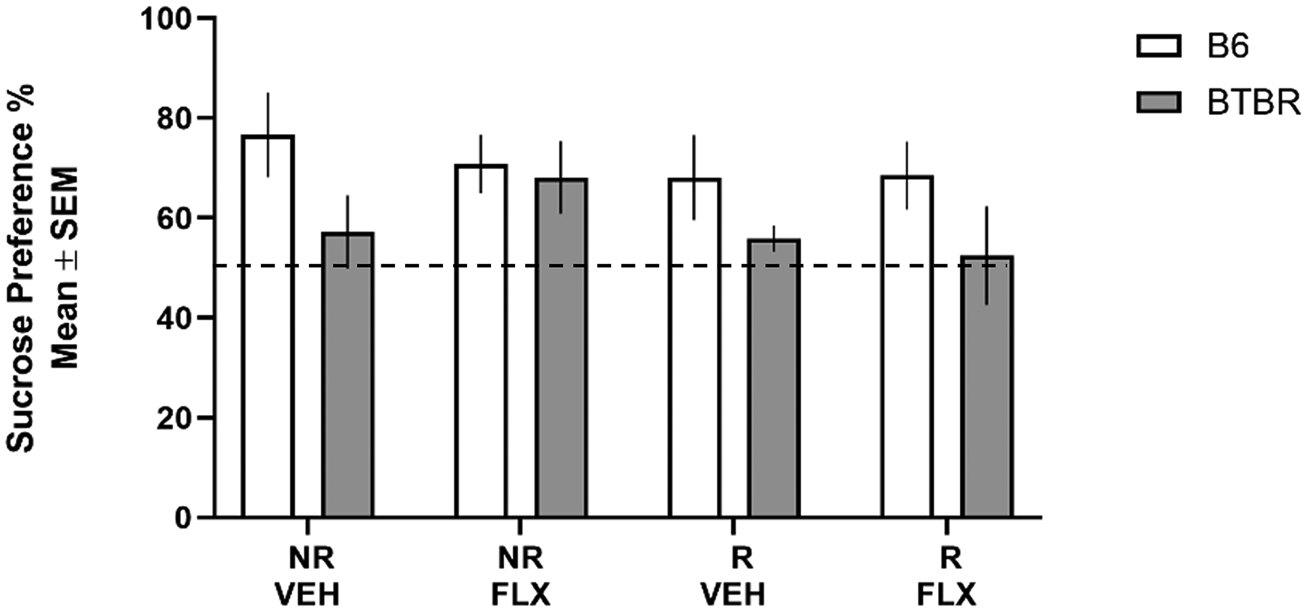
Sucrose Preference Test. B6 mice in all conditions exhibited a sucrose preference. BTBR mice in all conditions did not exhibit a sucrose preference. The ratio was calculated as the percentage of sucrose consumed during test day 3/ total volume of liquid intake during test day 3 × 100. NR/VEH = no restraint/vehicle; NR/FLX = no restraint fluoxetine; R/VEH = restraint/vehicle and R/FLX = restraint/fluoxetine.

A sucrose preference test was administered to determine if restraint stress and/or fluoxetine affected sucrose preference. As observed previously [21], B6 mice in the no restraint/vehicle condition exhibited a significant preference for the sucrose solution (~ 75 % sucrose solution consumed) [see Fig. 2]. B6 mice in all other conditions exhibited a similar preference level. Comparable to that shown in a past study [37], BTBR mice in the no restraint/vehicle condition did not exhibit a strong sucrose preference (~ 55 % sucrose solution consumed), as well as BTBR mice in the other conditions leading to a significant effect for strain (*F*_1,29_= 6.12, *p*= 0.02). There was not a significant effect for condition (*F*_3,29_= 1.37, *p*= 0.27) nor a significant strain x condition interaction (*F*_3,29_= 0.68, *p*= 0.57).

### 3.3. Self-Grooming behavior

**Fig. 3.**
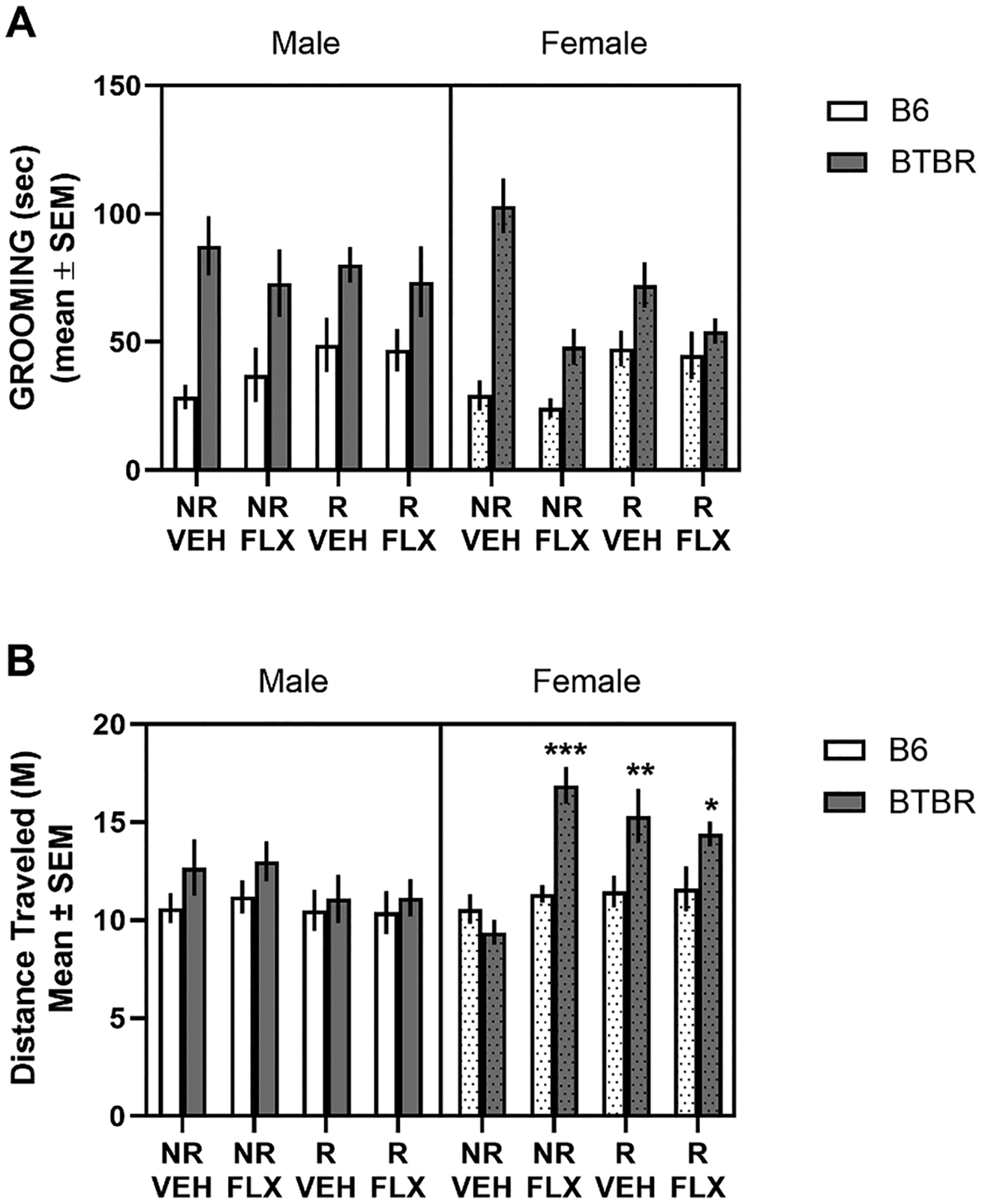
Grooming behavior and locomotor activity in the Self-Grooming Test. (A) Mean (± SEM) grooming duration. In the no restraint/vehicle condition, male and female BTBR mice had increased grooming behavior compared to that of B6 male and female mice. Prenatal manipulations reduced grooming duration in female BTBR mice. Prenatal stress conditions increased grooming in male and female B6 mice. (B) Mean (± SEM) locomotor activity during self-grooming test. Prenatal manipulations significantly increased distanced traveled in female BTBR mice. NR/VEH = no restraint/vehicle; NR/FLX = no restraint fluoxetine; R/VEH = restraint/vehicle and R/FLX = restraint/fluoxetine. *** *p*< 0.001 vs. female BTBR mice no restraint/vehicle; ** *p*< 0.01 vs. female BTBR mice no restraint/vehicle and * *p*< 0.05 vs. female BTBR mice no restraint/vehicle.

The effects of prenatal stress, fluoxetine or the combination on self-grooming behavior are shown in Fig. 3A. In the no restraint/vehicle condition, BTBR male and female mice exhibited greater grooming duration compared to that of B6 male and female mice. Prenatal exposure to stress, fluoxetine or the combination altered grooming duration differentially based on strain and sex. A three-way ANOVA with Kenward-Roger’s method revealed a significant main effect for strain (*F*1,17.92= 43.09, *p*< 0.0001) reflecting the greater grooming time in BTBR mice compared to that of B6 mice. No other factors were significant. However, there was a significant strain x condition interaction (*F*_3,_ 18.61= 5.08, *p* =0.009). Post-hoc tests indicated that BTBR mice in the no restraint/fluoxetine and restraint/ fluoxetine conditions groomed significantly less than in the no restraint/vehicle and restraint/vehicle conditions (*p*’s < 0.05). The reduced grooming in these conditions was strongest in the BTBR female mice in which grooming duration was reduced to half of that in the no restraint/vehicle condition. Further, B6 mice in the restraint/vehicle condition and restraint/fluoxetine condition groomed significantly more than B6 mice in the no restraint/vehicle condition (*p*’s < 0.05).

Fig. 3B illustrates the effects of prenatal stress and/or fluoxetine exposure on locomotor activity during the self-grooming test. Only female BTBR mice were affected by the prenatal manipulations with all conditions elevating locomotor activity compared to the control condition. This pattern in female BTBR mice was indicated by a significant strain x sex x condition interaction (*F*3,99.8= 3.04, *p*= 0.03). Post-hoc tests indicated that locomotor activity in female BTBR mice in the no restraint / vehicle condition was significantly less than that in the no restraint/ fluoxetine condition (*p*< 0.0001), restraint/ vehicle condition (*p*< 0.001) and restraint/ fluoxetine condition (*p*< 0.05).

### 3.4. Rearing behavior

**Fig. 4.**
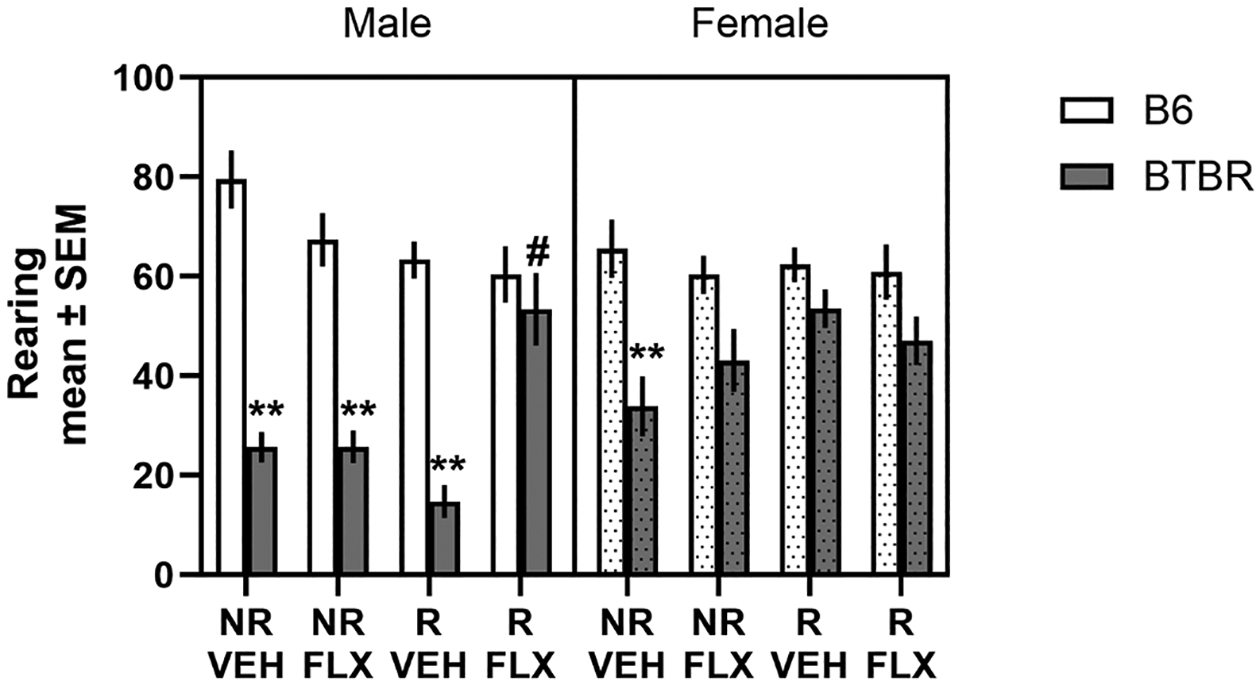
Effects of prenatal stress and fluoxetine on rearing behavior. Male BTBR mice in all conditions, except restraint stress/fluoxetine, exhibited significantly less rearing than male and female B6 in the no restraint/vehicle condition. Restraint stress/fluoxetine condition significantly increased rearing behavior in male BTBR mice compared to the other conditions in male BTBR mice. Female BTBR mice exhibited significantly less rearing compared to that of male and female B6 mice, while all prenatal manipulations increased rearing in female BTBR mice to a level intermediate between female BTBR and B6 mice in the no restraint/vehicle condition. NR/VEH = no restraint/vehicle; NR/FLX = no restraint fluoxetine; R/VEH = restraint/vehicle and R/FLX = restraint/fluoxetine. **p< 0.01 vs. male and female B6 no restraint/vehicle condition; # *p*< 0.05 vs. male BTBR mice no restraint/vehicle condition.

Rearing behavior was also measured during the self-grooming test (see Fig. 4). An analysis of the rearing behavior revealed a significant strain x sex x condition interaction (*F*3,99.7= 3.63, *p*= 0.02). Post-hoc tests revealed that both B6 male and female mice exposed to the no restraint/vehicle condition had significantly greater levels of rearing than that of BTBR male and female mice in the no restraint/vehicle condition (*p*’s < 0.01). In a comparable manner, BTBR male mice exposed to prenatal fluoxetine or prenatal stress exhibited significantly less than B6 male and female mice in the no restraint/vehicle condition (*p*’s < 0.01). The prenatal manipulations in both B6 male and female mice did not significantly change rearing behavior compared to the no restraint/vehicle condition in B6 male and female mice (*p*’s > 0.05). In BTBR female mice, prenatal exposure to fluoxetine, stress or the combination increased rearing behavior to a level that was not significantly different from that of B6 female mice in the no restraint/vehicle condition (*p*’s > 0.05), but also not significantly different from that of BTBR female mice in the no restraint/vehicle condition (*p*’s > 0.05). In BTBR male mice, prenatal exposure to the combination of fluoxetine and restraint stress significantly increased rearing behavior compared to that of BTBR male mice in the no restraint/vehicle condition (*p*< 0.05).

### 3.5. Jumping behavior

Jumping behavior results are shown in Table 1. All B6 and BTBR mice in the no restraint/vehicle condition showed low levels of jumping behavior with only 2 mice from each group displaying any jumping behavior. Prenatal fluoxetine, restraint stress or the combination did not affect jumping behavior in B6 mice, but did have selective effects in BTBR mice. An analysis revealed that there was a significant strain effect (*F*1,14.82= 23.72, *p*=0.0002). In addition, there was a significant condition x strain interaction (*F*3,15.75= 3.85, *p*=0.04). Post-hoc tests revealed that BTBR male mice in the restraint stress/fluoxetine condition exhibited a significantly greater amount of jumping behavior than B6 mice in all conditions (*p*’s < 0.01), as well as BTBR mice in the no restraint/vehicle condition (*p’s* < 0.01).

For BTBR female mice, the increased jumping behavior resulted in part from a significant increase in the proportion of mice exhibiting jumping behavior exposed to prenatal fluoxetine, stress and the combination compared to the no restraint/vehicle condition (p’s < 0.01). In BTBR male mice, the proportion of mice displaying jumping behavior significantly increased in the restraint/fluoxetine condition compared to the no restraint/vehicle condition (*p*< 0.01).

### 3.6. Elevated plus maze

**Fig. 5.**
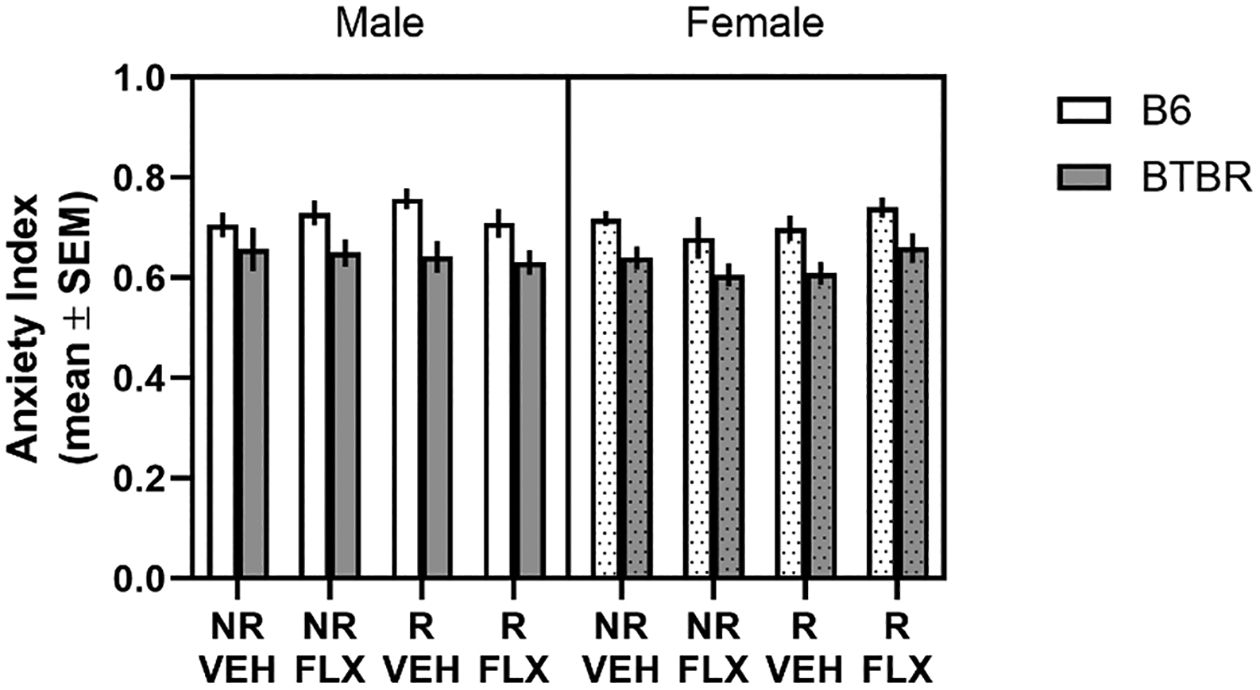
Effect of prenatal stress and/or fluoxetine on anxiety in the elevated plus-maze. B6 mice had a higher anxiety index than BTBR mice. Prenatal stress and/or fluoxetine did not affect the anxiety index in either strain. The anxiety index was calculated by first calculating the ratio of the open arm duration divided by the total of open, closed and center area durations plus the open arm entries divided by the total of open, closed and center area entries. This ratio was divided by 2. The resulting number was subtracted by 1. NR/VEH = no restraint/vehicle; NR/FLX = no restraint fluoxetine; R/VEH = restraint/vehicle and R/FLX = restraint/fluoxetine.

The effects of prenatal stress and/or fluoxetine on anxiety-related behavior was investigated in BTBR and B6 mice (see Fig. 5). Independent of sex and condition, BTBR mice showed a lower anxiety index score than that of B6 mice. This was reflected by a significant effect for strain (*F*1,14.86= 34.93, *p*= 0.00002). There were no other significant main effects or interactions.

### 3.7. Spatial acquisition and reversal learning with deterministic reinforcement

**Fig. 6.**
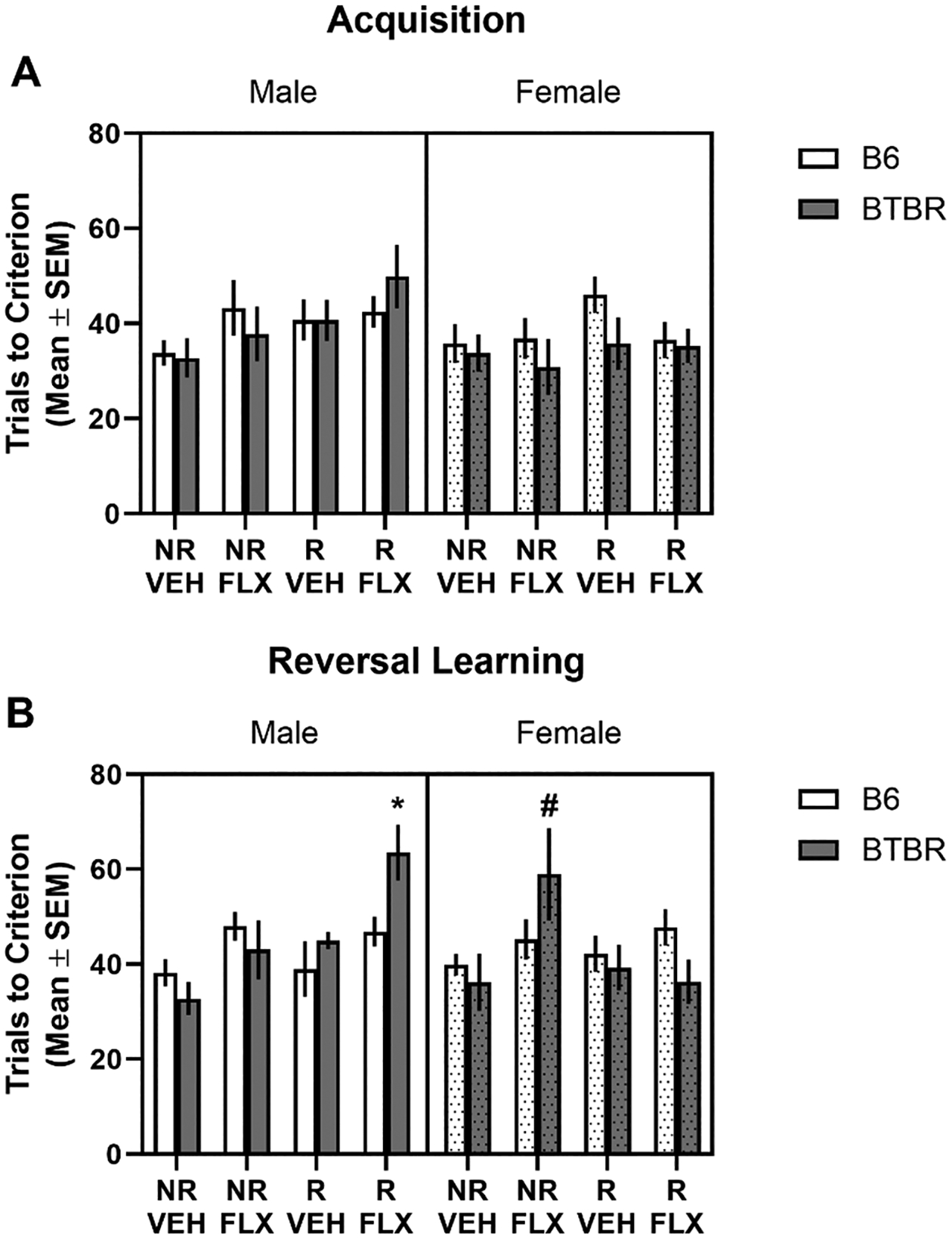
Effects of prenatal stress and/or fluoxetine exposure on spatial acquisition and reversal learning. (A) Mean (± SEM) trials to criterion in acquisition. Prenatal stress and/or fluoxetine exposure in B6 and BTBR mice did not impact trials to criterion in acquisition. (B) Mean (± SEM) trials to criterion in reversal learning. in C57BL/6 J male offspring. Combined prenatal stress and fluoxetine exposure significantly increased reversal learning trials in male BTBR mice while prenatal fluoxetine exposure alone increased reversal learning trials in female BTBR mice. NR/VEH = no restraint/vehicle; NR/FLX = no restraint fluoxetine; R/VEH = restraint/vehicle and R/FLX = restraint/fluoxetine. *p <0.05 vs. male BTBR mice no restraint/vehicle; # *p* <0.05 vs. female BTBR mice no restraint/vehicle.

Mice were tested on acquisition and reversal of a two-choice spatial discrimination. All groups achieved acquisition criterion in approximately 30–45 trials (see Fig. 6A). An analysis revealed there were no significant main effects or interactions for acquisition learning.

The results from reversal learning are shown in Fig. 6B There was a significant effect for condition (*F*3,15.77= 5.94, *p*= 0.006). In addition, there was a significant condition x sex interaction (*F*3,84.09= 3.23, *p*= 0.03). There was also a significant three-way interaction (*F*3,82.92= 5.18, *p*= 0.002). Post-hoc analyses revealed that BTBR male mice in the restraint stress/fluoxetine condition required significantly more reversal learning trials compared to all mice in the no restraint/vehicle condition (*p*’s < 0.05). BTBR female mice exposed to prenatal fluoxetine required significantly greater number of trials to achieve reversal learning criterion compared to that of BTBR female mice in the no restraint/vehicle condition (*p*< 0.05).

## 4. Discussion

Stress and SSRI exposure during pregnancy are two factors that may increase risk for autism in offspring, as well as contribute to heterogeneity in the disorder [2,3,5,6,11,41–43]. A recent study demonstrated that prenatal stress, fluoxetine or the combination led to stereotyped motor behaviors and behavioral flexibility deficits in a sex-dependent manner [21]. The present experiment investigated whether prenatal stress and/or SSRI exposure alters the behavioral phenotype in the BTBR mouse model of autism. The BTBR mouse exhibits a robust phenotype exhibiting social deficits [35,44–46], altered vocalizations [33,47,48], as well as repetitive motor behaviors and behavioral inflexibility [3,29, 30,49–51]. Other results from the BTBR mouse indicate down-regulation of autism risk genes, i.e. Scn1a, [28,52]; altered neurotransmitter levels similarly observed in ASD [52–54], as well as brain morphological changes comparable to that observed in ASD [55, 56]. Thus, the BTBR mouse has both brain and behavioral features that make it attractive as a mouse model of autism.

Besides measuring the effects of prenatal stress and fluoxetine on offspring, how these factors affect weight gain, litters and sucrose preference during pregnancy was determined. Dams in all groups exhibited significant weight gain across gestation. Although, restraint stress alone in BTBR mice significantly limited weight gain. However, the combination of restraint stress and fluoxetine did not affect weight gain. In contrast, the manipulations during pregnancy did not influence weight gain in B6 mice. This contrasts with past studies in which restraint stress during pregnancy reduced weight gain in B6 mice [21,57, 58]. The reduced amount of restraint stress used in this study compared to past studies is a likely explanation why weight gain was not affected in B6 mice.

Compared to a past study in which B6 mice received three 30-minute restraint stress sessions during gestations day 4–18 [21], we reduced the amount of restraint stress because initial results indicated that administering restraint stress even once daily reduced litter survivability in BTBR mice. Therefore, in this study we administered a single restraint stress session every other day starting on gestation day 4 through 18. This led to greater litter survivability compared to a single restraint stress session each day starting at gestation day 4 through 18 in BTBR mice. There was some variability in litter survivability among conditions and strain, but the analyses revealed there was no significant differences. The lowest litter survivability was observed in BTBR mice exposed to fluoxetine treatment which reduced litter survivability to 50 %. Despite this change in litter survivability, restraint stress, fluoxetine or the combination during pregnancy had no effects on litter size. This is comparable to that observed previously with restraint stress in B6 mice [21,59]. Overall, the manipulations during pregnancy did not produce significant effects on litter survivability or litter size.

In the sucrose preference test, restraint stress did not affect a sucrose preference in B6 mice. This contrasts with our previous study in which a greater amount of restraint stress during the same gestation period did reduce a sucrose preference in pregnant mice [21]. Restraint stress not affecting sucrose preference in the present study reflects this was a milder stress manipulation than used in the past [21]. As observed previously, fluoxetine treatment alone or combined with restraint stress led to a sucrose preference comparable to that observed in B6 controls. Unlike B6 controls, BTBR controls did not exhibit a strong sucrose preference. This is comparable to a past study in which BTBR mice did not show a sucrose preference [37]. Restraint stress, fluoxetine or the combination in BTBR mice did not significantly alter the behavioral pattern in the sucrose preference test. An examination of the BTBR mice revealed that a mutation in the inositol triphosphate receptor 3 gene underlies the taste dysfunction [37]. Taken together, the findings indicate that the amount of restraint stress, fluoxetine or the combination used in the present study do not affect sucrose consumption in either strain.

Prenatal stress, fluoxetine or the combination had long-term behavioral effects in offspring when tested as young adults. Specifically, the experiment investigated the effects on repetitive motor behaviors, anxiety and behavioral flexibility. In several mouse models of autism, including BTBR mice, elevated self-grooming is observed as part of the behavioral phenotype [31,34,38,60,61]. In the present study, both male and female BTBR mice in the no restraint/vehicle condition showed increased grooming compared to that of male and female B6 mice in the same condition. The prenatal manipulations did not affect grooming or activity in male BTBR mice. Female BTBR mice exposed prenatally to fluoxetine, stress or the combination showed reduced grooming behavior with concomitant elevated locomotor activity. Thus, prenatal exposure to fluoxetine, stress or combination in this context lead to hyperactivity in female BTBR mice which likely resulted in the reduced grooming for these conditions. In B6 mice, both male and female mice exhibited increased grooming in the restraint stress conditions. This is comparable to that observed in a previous study [21]. The findings in B6 mice suggest that prenatal stress can have long-term consequences in both male and female offspring by increasing repetitive motor behaviors.

In the self-grooming test, rearing and jumping behavior was also measured. Rearing behavior, in which an animal temporarily stands on its hind legs, is considered a form of exploratory behavior particularly when placed in a novel environment [62,63]. In the no restraint/vehicle condition, both male and female BTBR mice displayed less rearing than male and female B6 mice. This may not be surprising as BTBR mice spent greater time grooming. Prenatal stress or fluoxetine alone had no effect in male BTBR mice, but the combination significantly elevated rearing and jumping behavior. In female BTBR mice, the prenatal manipulations increased rearing behavior to levels that were between that of female B6 and BTBR mice in the no restraint/vehicle conditions. Further, the various stress and fluoxetine conditions increased jumping behavior in female BTBR mice. So prenatal stress, fluoxetine or combination in female BTBR mice did not simply increase locomotor activity, but additionally augmented exploratory behavior. Important to note, in almost all cases for mice that exhibited jumping behavior, a mouse would not only jump, but also climb on the rim of the testing cage and walk along the rim for a few seconds. Further, while the change in the behavioral pattern was similar in male and female BTBR mice, males were only affected by combined prenatal stress and fluoxetine. Female BTBR mice exhibited a behavioral change following all prenatal fluoxetine and stress conditions. Together, the behavioral changes may be related to dysfunction in the hippocampal formation, as this area plays an important role in exploring novel environments [62]. Further, the hippocampal formation is proposed to support behavioral inhibition in contexts that may be novel and/or anxiety-producing [64]. One possibility is that prenatal stress, fluoxetine and the combination altered hippocampal formation development in BTBR mice that reduced behavioral inhibition resulting in greater rearing and jumping behavior as observed in the self-grooming test. This interpretation is consistent with recent findings observed in B6 mice in which prenatal stress, fluoxetine or the combination reduces hippocampal synaptic plasticity with the combination most affecting synaptic plasticity in male mice and both prenatal manipulations, as well as the combination affecting synaptic plasticity in female mice [21].

Findings from the various measures in the self-grooming test revealed behavioral changes due to prenatal stress, fluoxetine or the combination that were distinct based on strain and sex. In B6 mice, prenatal stress alone or combined with fluoxetine increased grooming behavior in male and female offspring. In male BTBR mice, the prenatal manipulations did not affect grooming, but combined prenatal stress and fluoxetine increased both rearing and jumping behavior. Prenatal stress and fluoxetine exposure produced the greatest changes in female BTBR mice. Specifically, prenatal stress alone, fluoxetine alone and the combination reduced self-grooming behavior while concomitantly increasing locomotor activity, rearing and jumping behavior. Thus, the results suggest that maternal stress and anti-depressant treatment during prenatal development can have long-term consequences in repetitive motor behavior, locomotion and exploratory behavior.

Repetitive and restricted behaviors in ASD are not limited to elevated stereotyped motor behaviors, but also represent circumscribed interests, rituals, and behavioral inflexibility [65–68]. The study further examined whether exposure to maternal stress and/or fluoxetine exposure during prenatal development had effects on behavioral flexibility in offspring. We employed a spatial discrimination test that included an acquisition and reversal learning test. Past experiments demonstrated that BTBR mice are not impaired in acquiring a spatial discrimination with probabilistic reinforcement but are impaired on spatial reversal learning with probabilistic reinforcement [29,30,49,69]. In addition, BTBR mice are not impaired in acquiring or reversal of a spatial discrimination when the reinforcement is deterministic (one choice always correct and one choice always incorrect) [69]. Because BTBR mice are not impaired on acquiring or reversing a spatial discrimination with deterministic reinforcement, we wanted to determine whether prenatal stress, fluoxetine or the combination would induce a deficit. No impairments were observed in acquisition for BTBR and B6 mice exposed to the prenatal manipulations. This is comparable to past studies demonstrating that prenatal stress and fluoxetine exposure does not affect learning in offspring [70,71]. However, combined prenatal stress and fluoxetine impaired reversal learning in male BTBR mice and prenatal fluoxetine exposure impaired reversal learning in female BTBR mice. Thus, in a behavioral flexibility test in which BTBR mice do not show deficits [69], the prior exposure to fluoxetine or combined restraint stress and fluoxetine during prenatal development could produce behavioral flexibility impairments in either male and female BTBR mice.

The broader interpretation of the behavioral results is that prenatal stress and/or SSRI exposure alters neurodevelopment starting in the prenatal period that has long-lasting consequences which continue through adulthood. An alternative explanation is that restraint stress and/or fluoxetine treatment in pregnant dams changed maternal behavior that led to behavioral changes in offspring. The present study did not measure maternal behavior. One study using CD1 mice found that stress exposure during pregnancy affected maternal behavior [58], but another study with B6 mice, as used in the present experiment, found that restraint stress, fluoxetine or the combination did not change maternal behavior [71]. Similarly, prenatal stress in rats did not affect maternal behavior [72]. In addition, in the current study, restraint stress, fluoxetine or the combination did not affect sucrose preference in B6 mice that might give an indication that maternal behavior was not altered. In BTBR mice, restraint stress alone did reduce body weight gain, but the restraint stress alone condition did not produce any selective behavioral changes in male offspring or different from the other prenatal manipulation conditions in female offspring. This might suggest BTBR dams did not have altered maternal behavior. Although a change in maternal behavior cannot be completely ruled out the findings in B6 mice from the present study and based on past results [71,72] suggest that a change in maternal behavior is not a likely explanation for the observed behavioral changes in offspring. Systematically examining this in future studies is important to thoroughly address this issue.

Another possible explanation for the behavioral changes observed following prenatal stress, fluoxetine and/or the combination is that the prenatal manipulations increased anxiety that subsequently changed behavioral patterns in the self-grooming test and spatial discrimination test. However, no differences in anxiety as measured in the elevated-plus maze across the various experimental conditions occurred in both mouse strains. Thus, the findings from the elevated plus-maze test further suggest that changes observed in self-grooming and reversal learning tests cannot be simply explained by an overall increase in anxiety-like behavior but are more likely due to long-term changes in neural systems that support behavioral flexibility and regulate expression of stereotyped and exploratory behaviors.

An altered excitation/inhibition balance in the brain has been proposed to underlie an autism phenotype [73,74]. Prenatal stress or SSRI exposure in rodents can lead to an excitatory/inhibition imbalance in frontal cortex, striatum and hippocampus [72,75–78] Further, these prenatal manipulations can lead to differential brain and behavioral outcomes based on sex [78]. Related to repetitive behaviors and restricted interests, frontal-striatal systems are proposed to support the expression of stereotyped motor behaviors, as well as behavioral flexibility [79–81]. Because the BTBR mouse shows altered glutamate signaling in this brain circuity [54,82], one possibility is that prenatal manipulations exacerbated an excitation/inhibition imbalance in frontal-striatal circuits that contributed to altered motor behaviors and behavioral flexibility deficits in BTBR mice. As discussed above, the hippocampus may also be affected by these prenatal experiences producing an excitation/inhibition imbalance that changes activity and exploratory behavior. Taken together, prenatal stress and/or fluoxetine may alter development of multiple neural systems producing an excitatory/inhibitory imbalance in multiple brain regions leading to a complex behavioral phenotype.

## 5. Conclusions

A significant challenge in understanding and treating ASD is the heterogeneity that occurs at multiple levels ranging from genetic heterogeneity to behavioral heterogeneity [83]. Another concern in ASD is the comorbidity with other disorders that may limit adaptive behavior [84,85]. Epilepsy, gastrointestinal dysfunction and attention disorder with hyperactivity (ADHD) are other disorders that commonly occur with ASD [86–88]. Heterogeneity and co-morbidity in ASD may occur, in part, from an interaction of environmental and genetic risk factors [22,23]. In the present experiment, the various prenatal manipulations did not broadly produce the same behavioral effects in both mouse strains but led to fairly distinct behavioral changes based on strain and sex. Importantly, the findings from the present experiment demonstrate how prenatal stress, fluoxetine or the combination can produce distinct phenotypes in a mouse model of autism suggesting these prenatal experiences may contribute to the heterogeneity observed in ASD. This overall pattern of results suggests that prenatal experiences, i.e. stress and fluoxetine exposure, may interact differentially based on genetics and sex to produce various behavioral phenotypes involving ASD that include other conditions frequently associated with ASD, i.e. ADHD. Thus, prenatal manipulations combined with various rodent models of autism may provide novel insights into the neuropathophysiology underlying particular autism phenotypes, as well as enhance the translation of effective treatments.

## Supplementary Material

1

Supplementary material associated with this article can be found, in the online version, at doi:10.1016/j.physbeh.2025.114891.

## Figures and Tables

**Table 1:** Summary of jumping behavior in B6 and BTBR mice across the various restraint stress and fluoxetine conditions. Combined prenatal stress and fluoxetine significantly increased jumping behavioral in male and female BTBR mice compared to B6 and BTBR mice in the no restraint/vehicle condition. Prenatal fluoxetine, stress or the combination significantly increased the number of female BTBR mice who displayed jumping behavior and the combination of prenatal stress and fluoxetine significantly increased the number of mice who jumped in male BTBR mice.

	Group mean (SEM)	Range	Number of mice jumping
*No Restraint/Vehicle*			
**B6 male**	0.50 (0.42)	0–5	2/12
**BTBR male**	0.56 (0.38)	0–3	2/9
**B6 female**	0.36 (0.26)	0–3	2/11
**BTBR female**	0.33 (0.22)	0–2	2/12
*No Restraint/Fluoxetine*			
**B6 male**	0.20 (0.13)	0–1	2/10
**BTBR male**	0.67 (0.58)	0–7	2/12
**B6 female**	0.25 (0.16)	0–1	2/10
**BTBR female**	6.25 (3.78)	0–32	5/8**
*Restraint/Vehicle*			
**B6 male**	0.33 (0.24)	0–2	2/9
**BTBR male**	2.33 (1.34)	0–15	4/12
**B6 female**	0.20 (0.13)	0–1	2/10
**BTBR female**	3.75 (1.02)	0–12	9/12**
*Restraint/Fluoxetine*			
**B6 male**	0.11 (0.11)	0–1	1/9
**BTBR male**	4.70 (1.56)**	0–15	7/10**
**B6 female**	0.18 (0.12)	0–1	2/11
**BTBR female**	3.45 (0.99)**	0–10	8/11**

** *p*< 0.01 vs B6 and BTBR mice no restraint/vehicle.

## Data Availability

Data will be made available on request.
