# Supplementary material for: Prenatal stress and fluoxetine exposure in BTBR and B6 mice differentially affects autism-like behaviors in adult male and female offspring
Source: Physiol Behav. Author manuscript; Available in PMC 2026 May 14. (PMC13174746; doi:10.1016/j.physbeh.2025.114891)
Supplement: 1 [file NIHMS2173342-supplement-1.pdf]

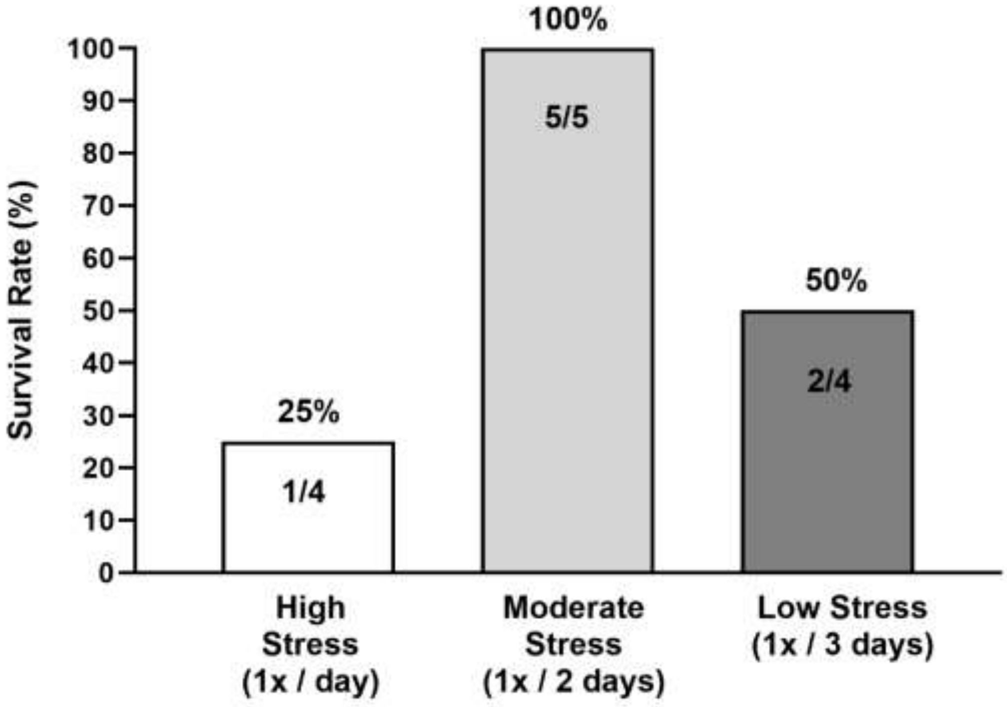

Supplementary Figure 1 Caption. Effect of different restraint stress protocols on litter survival in BTBR mice. High stress condition led to 25% litter survival rate while moderate stress condition led to 100% litter survival rate.
